# Cognitive Empathy and Longitudinal Changes in Temporo-Parietal Junction Thickness in Schizophrenia

**DOI:** 10.3389/fpsyt.2021.667656

**Published:** 2021-05-14

**Authors:** Tatiana Karpouzian-Rogers, Derin Cobia, Julie Petersen, Lei Wang, Vijay A. Mittal, John G. Csernansky, Matthew J. Smith

**Affiliations:** ^1^Department of Psychiatry and Behavioral Sciences, Northwestern University Feinberg School of Medicine, Chicago, IL, United States; ^2^Mesulam Center for Cognitive Neurology and Alzheimer's Disease, Northwestern University Feinberg School of Medicine, Chicago, IL, United States; ^3^Department of Psychology and Neuroscience Center, Brigham Young University, Provo, UT, United States; ^4^Department of Psychiatry and Behavioral Health, The Ohio State University Wexner Medical Center, Columbus, OH, United States; ^5^Department of Psychology, Northwestern University, Evanston, IL, United States; ^6^School of Social Work, University of Michigan, Ann Arbor, MI, United States

**Keywords:** schizophrenia, cognitive empathy, temporoparietal junction, neuroimaging, emotional perspective-taking

## Abstract

**Objective:** Deficits in cognitive empathy are well-documented in individuals with schizophrenia and are related to reduced community functioning. The temporoparietal junction (TPJ) is closely linked to cognitive empathy. We compared the relationship between baseline cognitive empathy and changes in TPJ thickness over 24 months between individuals with schizophrenia and healthy controls.

**Methods:** Individuals with schizophrenia (*n* = 29) and healthy controls (*n* = 26) completed a cognitive empathy task and underwent structural neuroimaging at baseline and approximately 24 months later. Symmetrized percent change scores were calculated for right and left TPJ, as well as whole-brain volume, and compared between groups. Task accuracy was examined as a predictor of percent change in TPJ thickness and whole-brain volume in each group.

**Results:** Individuals with schizophrenia demonstrated poorer accuracy on the cognitive empathy task (*p* < 0.001) and thinner TPJ cortex relative to controls at both time points (*p* = 0.01). In schizophrenia, greater task accuracy was uniquely related to less thinning of the TPJ over time (*p* = 0.02); task accuracy did not explain changes in left TPJ or whole-brain volume. Among controls, task accuracy did not explain changes in right or left TPJ, or whole-brain volume.

**Conclusions:** Our findings suggest that greater cognitive empathy may explain sustained integrity of the right TPJ in individuals with schizophrenia, suggesting a contributory substrate for the long-term maintenance of this process in psychosis. Cognitive empathy was not related to changes in whole-brain volume, demonstrating the unique role of the TPJ in cognitive empathy.

## Introduction

Social cognition broadly refers to a psychological construct that describes how one thinks about themselves in relation to others and the processes involved in social interactions (1). One component of social cognition is empathy, which is the ability to be sensitive to and understand the mental state of others (2). More specifically, this component can be separated into two subsystems reflecting basic emotion-contagion and perspective-taking (3). The latter subsystem, termed cognitive empathy, requires the ability to infer or understand the perspective and emotions of others, and utilizes higher-order cognitive processes such as cognitive flexibility (4, 5). For example, if a friend has experienced a loss, one may be able understand their thoughts or emotions by imagining oneself in this situation, despite never having experienced loss personally.

Individuals with schizophrenia demonstrate difficulties in several aspects of social cognition, including misperception of social cues, poor mentalizing or perspective-taking ability, and less accurate emotion monitoring (6, 7). Subsequent to weaknesses in social cognitive abilities, many individuals with schizophrenia experience strained social relationships, which can contribute to impairments in day-to-day functioning (8). A meta-analysis by Fett et al. (9) determined that social cognition may be more highly related to aspects of functioning than general cognition. Further, deficits in cognitive empathy have been associated with lower functional capacity, social competence, and social attainment in schizophrenia after accounting for general cognition and psychopathology (10–13). Additionally, interventions targeted toward improving aspects of social cognition may lead to improvements in social functioning (14). For example, an intervention designed to address multiple components of social cognition in individuals with psychosis led to significant improvement in social cognitive abilities (15, 16). While current research remains limited, there is some initial evidence that social skills training may improve real-world functioning (7). Given the link between cognitive empathy and functional abilities in schizophrenia, understanding the mechanisms underlying this fundamental ability is of great importance.

The brain networks underlying social cognitive processes are complex and may work in tandem during social interactions. Specifically, Van Overwalle and Baetens (17) describe a mirror system, which is a lower-level system that allows one to sense and recognize the goal of another's action and match it to a representation of our own, while the higher-order mentalizing system helps one to infer the thoughts and feelings of others and understand another's beliefs as separate from our own through use of “social intelligence.” According to the authors, the former system consists of the anterior intraparietal sulcus, premotor cortex, and superior temporal sulcus, while the latter includes the precuneus, medial prefrontal cortex (mPFC), and temporo-parietal junction [TPJ; (17)]. Moreover, several studies indicate the TPJ is uniquely related to the higher order mentalizing system among healthy controls (18–20). In contrast, individuals with schizophrenia demonstrate aberrant neural activation patterns in the TPJ while performing a cognitive empathy task (21) and other mentalizing tasks (6), with the most common finding being reduced activation, suggesting greater neural resources are needed for intact mentalizing. A review by Eddy (22) underscored the importance of the TPJ in the interface between processing sensory experiences and understanding one's internal mental or motivational state, and that this system may be compromised in neuropsychiatric populations, including schizophrenia.

In addition to abnormal functional activity, individuals with schizophrenia demonstrate reduced cortical thickness in regions associated with cognitive empathy, including the TPJ (23–25). Notably, a prior study observed greater thickness of the TPJ was associated with stronger cognitive empathy in healthy controls, though this relationship was not observed in individuals with schizophrenia (26). However, few studies have examined longitudinal changes in neuroanatomical regions associated with social cognition, and how this may be related to cognitive empathy. This is of particular interest given the increased rates of cortical thinning in schizophrenia over time, and its relationship with functional and symptomatic outcomes (27). Interestingly, neuroanatomical substrates may be plastic over time, such that experience and behavior may alter neural circuits across psychiatric disorders (28). Thus, the present study examined whether accuracy on a cognitive empathy task explained significant variation in changes in TPJ thickness over 24 months in individuals with schizophrenia and healthy controls. We predicted that in individuals with schizophrenia, there would be greater thinning of the TPJ over time and that stronger baseline cognitive empathy abilities would be associated with less thinning of the TPJ in this group.

## Methods

### Participants

Individuals with schizophrenia (*n* = 29) and healthy controls (*n* = 26) were recruited as part of a larger observational study investigating social cognition in schizophrenia (12, 26); there were no interventions as part of the study. All participants were between the ages of 18–45. Participants were excluded if they: (1) met Diagnostic and Statistical Manual of Mental Disorders-4th Edition [DSM-IV; (29)] criteria for substance abuse or dependence within the past 6 months; (2) had a severe medical condition; or (3) had sustained a significant head injury. Controls were additionally excluded if they had a lifetime history of a DSM-IV Axis I disorder or a first-degree relative with a psychosis spectrum disorder. The Institutional Review Board at Northwestern University approved all study procedures. All research participants provided written informed consent prior to study enrollment.

### Study Measures

#### Demographic and Clinical Measures

Demographic and clinical characteristics were assessed using the Structured Clinical Interview for DSM-IV (SCID; 26), which was administered by Master- and PhD-level research staff. Diagnosis was validated from a consensus between the SCID and a semi-structured interview with a study psychiatrist. Antipsychotic medication dosages were converted into chlorpromazine equivalents using a standardized method (30). Psychopathology was assessed in schizophrenia participants using the global ratings from the Scale for the Assessment of Positive Symptoms (31) and the Scale for the Assessment of Negative Symptoms (32). Participant demographics, as well as clinical characteristics of the schizophrenia group, are listed in Table 1. There were no group differences in age, sex, or race (all *p*-values > 0.05).

**Table 1:** Demographic and clinical characteristics of study sample.

	**CON (*n* = 26)**	**SCZ (*n* = 29)**	**Test statistic (*t* or χ^**2**^)**
Age, mean (SD)	31.3 (8.5)	33.7 (6.5)	1.15
Sex, M:F	16:10	17:12	0.05
Race, Ca:AA:Other)	14:8:4	13:11:5	0.46
Years of education, mean (SD)	15.6 (2.3)	13.0 (1.7)	4.7^*^
Parental SES, mean (SD)	26.9 (10.6)	23.2 (11.7)	1.2
Duration of illness, Mean years (SD)	–	15.4 (7.1)	–
Chlorpromazine equivalent (mg), Mean dose (SD)	–	355.0 (228.1)	–
SAPS global ratings, mean (SD)			
Hallucinations	–	2.6 (2.1)	–
Delusions	–	3.0 (1.8)	–
Bizarre behavior	–	1.7 (1.9)	–
Positive formal thought disorder	–	2.1 (1.6)	–
SANS global ratings, mean (SD)			
Affective flattening	–	3.3 (1.3)	–
Alogia	–	2.6 (1.6)	–
Avolition	–	3.3 (1.4)	–
Anhedonia	–	2.9 (1.4)	–
Attention	–	2.0 (1.9)	–

* *p < 0.05*.

#### Cognitive Empathy Task

**Figure 1:**
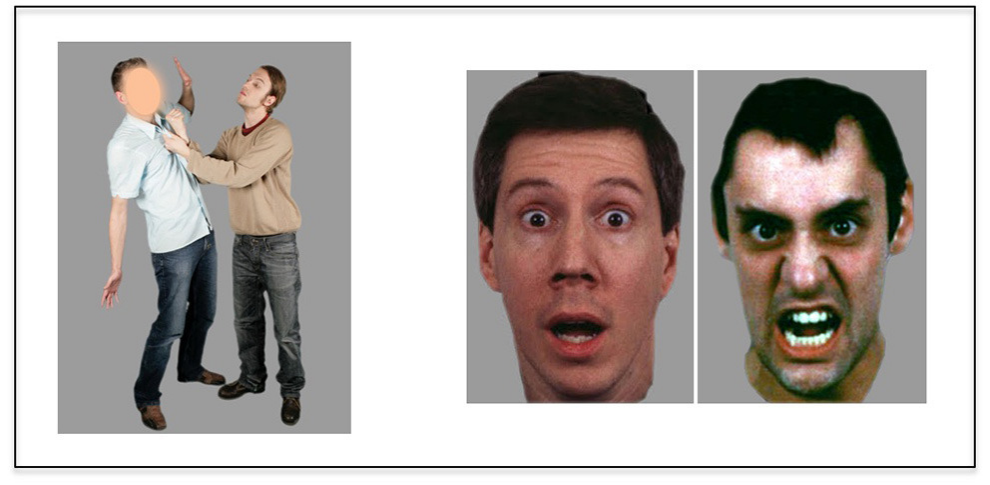
Schema of emotion perspective-taking task. Participants are asked to choose which emotional expression (shown on the right) fits with the masked face (shown on the left).

Cognitive empathy was assessed using the Emotional Perspective-Taking (EPT) task that was adapted into English (12) from the original version developed in Germany (33). In this task, participants were presented with scenes depicting social interactions between two Caucasian individuals (male or female), with one of the faces covered by a mask (Figure 1). After the scene was displayed for 4 s, two faces displaying different emotions were presented and participants were asked to choose which face best characterized the masked individual. Emotions included fear, anger, sadness, disgust, happiness, or neutrality. Participants responded using a response box, with left or right responses corresponding to the face on the left or right side of the screen. The task consisted of 60 trials, with 10 stimuli per emotion condition. The location of the responses was balanced across trials. Accuracy rates were calculated by dividing the total number of correct trials by the total number of trials completed.

### MRI Acquisition and Image Processing

MR scanning was performed at both baseline and follow-up visits (~24 months post-baseline) on a 3T TIM Trio system (Siemens Medical Systems) at Northwestern University's Center for Translational Imaging. Anatomical MRI was collected using a high-resolution 3D T1-weighted MPRAGE sequence optimized for gray-white contrast (echo time (TE) = 3.16 ms, repetition time (TR) = 2,400 ms, 1 × 1 × 1 mm voxels, time = 8.09 min).

All MR images were processed using the FreeSurfer (FS) toolkit release 5.3.0 (34) and manually edited according to established guidelines (35). Embedded FS longitudinal algorithms that reduce intra-subject morphological variability inherent in image processing were used for reconstruction of the cortical surface (36). Based on prior literature support (see above), the temporo-parietal junction (TPJ) was identified a priori as a key region involved in the regulation of cognitive empathy. Definition of this area was based on the supramarginal gyrus ROI derived from a default FS parcellation scheme (37) that was mapped across subjects using a non-linear surface registration procedure (38). Cortical thickness values (in mm) of the ROI from each subject in left and right hemispheres at both time points were calculated using embedded FS algorithms. Additionally, whole-brain volume was calculated in mm^3^ as the total volume of all brain voxels located above the cerebellar tentorium for each subject (i.e., “SupraTentorial” value computed by FS morphometry statistics). Whole-brain volume was calculated in lieu of global cortical thickness due to the regional differences that may affect global cortical thickness.

### Data Analysis

Differences in age, years of education, and parental socioeconomic status [SES; (39)] were assessed using independent samples *t*-tests, and differences in race and gender were assessed using chi-squared tests. In order to examine differences in performance on the EPT task between groups, independent samples *t*-tests were used with accuracy percentage and reaction times as dependent variables. Prior to imaging analysis, a ~99% (3 SD) winsorization of cortical thickness values was conducted per group in order to reduce the influence of spurious outliers (40). Using this method, there were no outliers. We then assessed differences in TPJ thickness over time (i.e., baseline to 24 months) by conducting a Repeated-Measures Analysis of Variance (RM-ANOVA), using group as a between-subject factor, with time and hemisphere (left or right) as within-subject factors. Lastly, we conducted a linear regression model using cognitive empathy task accuracy as variable to explain percent change in TPJ thickness separately per group. For this model, the independent variable (TPJ thickness percent change) was calculated as a symmetrized percent change (*spc*) score that estimates rate of change with respect to average thickness across timepoints (41) using the following formula: [(thickness at time 2 – thickness at time 1)/(months between scans)]/0.5 * (thickness at time 2 + thickness at time 1). In order to determine if changed in TPJ thickness was uniquely related to cognitive empathy task accuracy, we also calculated a *spc* score for whole-brain volume using the same procedure and conducted a linear regression using cognitive empathy as a predictor for changes in whole-brain volume.

## Results

### Group Differences on Cognitive Empathy Task

Controls and individuals with schizophrenia differed in accuracy on the emotional perspective-taking task [*t*_(53)_ = 4.75, *p* < 0.001], such that controls had a higher accuracy (mean = 0.85; SD = 0.07) than schizophrenia participants (mean = 0.73; SD = 0.11). Additionally, controls and schizophrenia participants differed in reaction time [*t*_(53)_ = 2.33, *p* = 0.02], such that controls were faster in responding (mean = 1,415 ms; SD = 300 ms) than schizophrenia participants (mean = 1,643 ms; SD = 411 ms).

### Group Differences in TPJ Thickness Over Time

A RM-ANOVA examining changes in TPJ thickness over time revealed an overall main effect of group on thickness, such that TPJ thickness in controls was greater than schizophrenia participants [*F*_(1, 53)_ = 7.00, *p* = 0.01; Table 2]. There were no significant main effects for time (*p* = 0.45) or hemisphere (*p* = 0.06), and no significant interactions for group by time, group by hemisphere, or group by-time-by-hemisphere interaction effects (all *p* > 0.5).

**Table 2:** Means (standard deviations) of TPJ thickness (mm) in controls and schizophrenia participants at baseline and follow-up.

		**CON (*n* = 26)**	**SCZ (*n* = 29)**	**Group effect**	**Time effect**	**Group X time**
Baseline	Right TPJ	2.68 (0.14)	2.58 (0.14)	*F* = 7.00^*^	*F* = 0.57	*F* = 1.03
	Left TPJ	2.63 (0.15)	2.56 (0.11)			
Follow-up	Right TPJ	2.67 (0.14)	2.57 (0.15)			
	Left TPJ	2.64 (0.13)	2.55 (0.12)			

* *p < 0.05. TPJ, temporo-parietal junction; CON, healthy controls; SCZ, individuals with schizophrenia*.

### Baseline Cognitive Empathy Task Performance as a Predictor for Changes in TPJ Thickness

**Figure 2:**
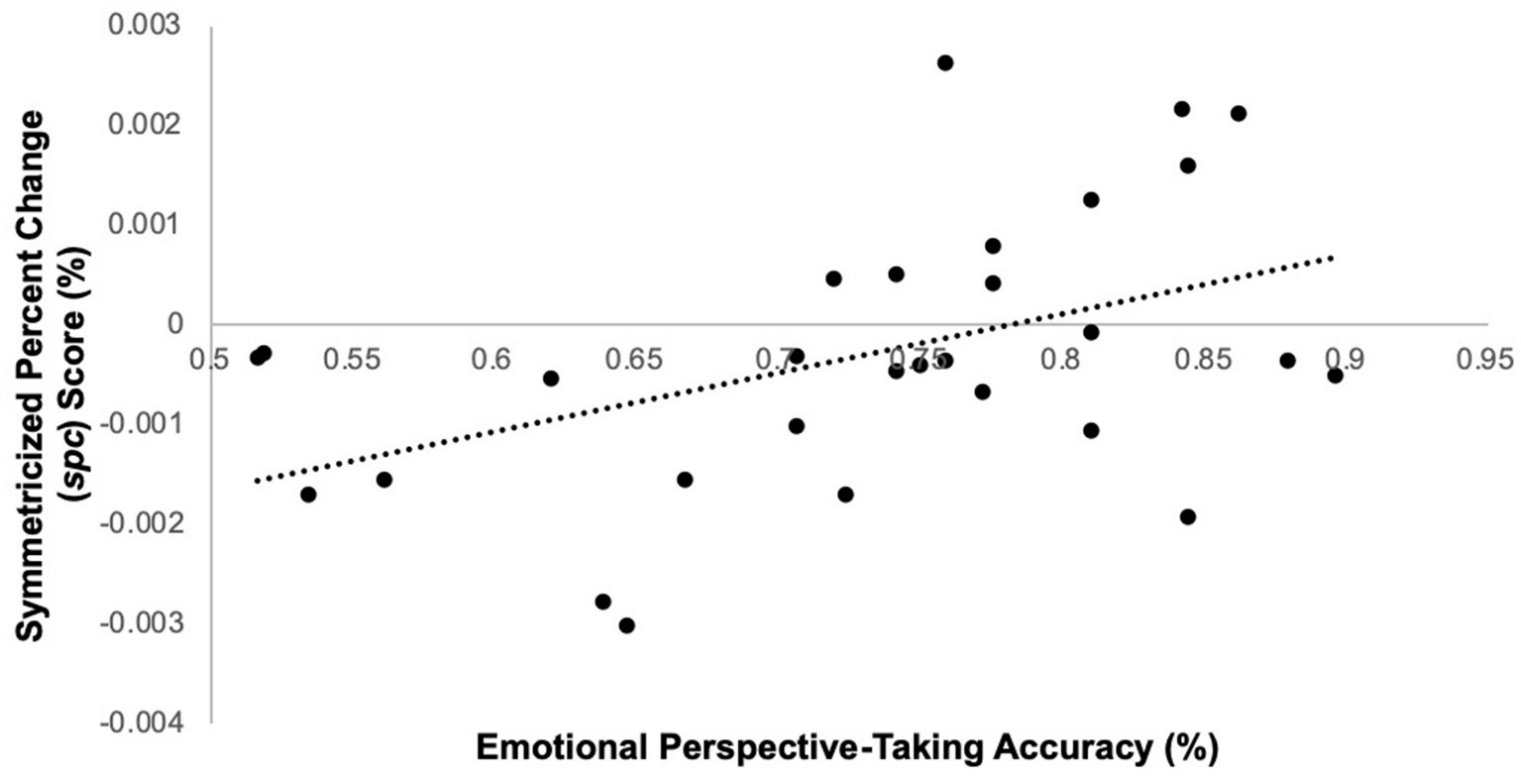
Emotion perspective-taking performance predicts symmetrized percent change score in right TPJ in schizophrenia participants only.

Among schizophrenia participants, task performance predicted right TPJ *spc* scores, such that lower accuracy predicted lower *spc* scores [i.e., greater TPJ thinning over time; *F*_(1, 28)_ = 6.9, *p* = 0.01; β = 0.45] (Figure 2); cognitive empathy accuracy did not significantly predict left *spc* scores [*F*_(1,28)_ = 0.57, *p* = 0.46; β = −0.14]. Among controls, accuracy did not significantly predict right [*F*_(1, 25)_ = 0.46, *p* = 0.50; β = 0.14] or left *spc* scores [*F*_(1, 25)_ = 0.68, *p* = 0.42; β = −0.17]. Lastly, cognitive empathy accuracy did not predict whole brain volume change in either schizophrenia participants [*F*_(1, 25)_ = 0.66, *p* = 0.43; β = 0.15] or controls [*F*_(1, 25)_= 0.30, *p* = 0.59; β = 0.11].

## Discussion

The purpose of this study was to investigate the relationship between baseline cognitive empathy performance and longitudinal changes in a key brain region underlying cognitive empathy, the temporoparietal junction, in individuals with schizophrenia and healthy controls. Consistent with prior studies (12, 26), our findings suggest that individuals with schizophrenia had a lower accuracy on the cognitive empathy task than controls. Also, we observed that the TPJ was thinner at both time points in individuals with schizophrenia compared to controls, though neither group experienced a significant change in thickness over the 24-month interval. Among the individuals with schizophrenia, but not the controls, we observed that greater accuracy on the cognitive empathy task was related to less thinning of the right TPJ over time, and this relationship was not observed in the left TPJ nor with whole-brain volume.

Abnormal thinning of the cortex is a characteristic feature of schizophrenia (42), with implications for disrupted cognitive processes (43). Individuals with chronic schizophrenia may show particularly increased cortical thinning in prefrontal and temporal cortices (25), and greater rates of thinning may be related to negative symptoms in schizophrenia (44). Several studies have demonstrated specific abnormalities in the TPJ in schizophrenia (42, 45), while some *ex vivo* work in a smaller sample noted limited changes (46). In the present study, although there were significant differences in TPJ cortical thickness between individuals with schizophrenia and controls, there were no significant longitudinal changes. While sample size may have contributed to this lack of finding, it is not entirely surprising given longitudinal studies of cortical thickness in schizophrenia are mixed on progressive deterioration of the TPJ, with some demonstrating loss (47) and others not (27). Given the significant biological heterogeneity that exists in schizophrenia (48), it is likely our sample consisted primarily of individuals who demonstrated subtle or variable degrees of change in TPJ thickness over the 2-year period of our study.

The main finding that greater cognitive empathy accuracy at baseline predicts reduced cortical thinning over time in individuals with schizophrenia suggests a potential resilience mechanism as it pertains to the progressive features of the illness. Interestingly, this relationship did not appear at a global level, indicating this is not a general feature, but rather one that is specific to the right TPJ. Furthermore, a large meta-analysis concluded that the right TPJ is a central brain region that infers the goals of others, and that this region is not just engaged by the orientation to people or actions, but rather related to higher-order social cognitive process (17). This function differs from other neural regions involved in cognitive empathy such as the mPFC, which appears to be a substrate for the attribution of more stable traits about one's self or others (49). Additionally, functional neuroimaging studies in healthy controls have demonstrated distinct neural networks that implicate the ventral TPJ for cognitive based empathy network [i.e., theory of mind; (50)], further demonstrating the unique role of the TPJ in cognitive empathy. More broadly, there may be disruptions in connectivity between the right TPJ and other neural regions that support both social cognitive and general cognitive functions, including the dorsolateral prefrontal cortex, cingulate cortex, and insula in individuals with schizophrenia (51). Further, the right hemisphere is necessary for social communication, including understanding tone of voice or processing alternate meanings of statements; prior studies have demonstrated that individuals with schizophrenia have difficulty on assessments of these right hemisphere functions, therefore making it difficult to understand the intent of others (52). Importantly, findings of right hemisphere and rTPJ dysfunction during social cognitive tasks may be observed in siblings of individuals with schizophrenia, raising questions about the heritability of rTPJ functioning and social cognition (53, 54). These studies, in parallel with our findings, demonstrate not only the unique role of the right TPJ in cognitive empathy, but also suggest that compromised functioning of this region may lead to disrupted functioning of other networks that underlie social and general cognitive abilities.

Several reasons may account for stronger baseline cognitive empathy abilities predicting reduced cortical thinning in the right TPJ among individuals with schizophrenia. First, one hypothesis is that individuals with schizophrenia who regularly engage cognitive empathy skills may invoke biological mechanisms that protect against greater rates of cortical thinning. It has been hypothesized that increased cognitive stimulation may be related to brain reserve via plasticity, or the ability of the brain to change and adapt both structurally and functionally in response to different experiences (55). A prior study demonstrated a potential association between functional connectivity and structural integrity of the rTPJ, suggesting a relationship between structure and function of the TPJ during theory of mind processes (56). While the relationship between greater utilization of empathy skills and brain reserve has not been investigated at an experimental level, this study may have clinical implications for remediating reduced cognitive empathy abilities experienced by individuals with schizophrenia (57) and preservation of brain structure. Alternatively, our findings can be interpreted as reduced accuracy predicting greater rates of thinning in individuals in schizophrenia. This explanation somewhat parallels previous findings of the relationship between greater rates of thinning or volume loss and worse cognitive or symptom outcomes in schizophrenia spectrum disorders (27, 58–60). However, this interpretation has been debated (61). Thus, it is also possible that thinning of the TPJ is already occurring in individuals with schizophrenia, as evidenced by overall group differences in cortical thickness, and that our behavioral marker of cognitive empathy is highlighting individuals with less distinct degradation. Further, preexisting TPJ thinning due to neurodevelopmental processes may make one more vulnerable to further thinning due to less use of this structure during social interactions. An alternative explanation is that our sample may include a distinct subgroup of individuals with schizophrenia with intact cortical thickness who also demonstrate preserved cognitive empathy abilities, while another subgroup of participants may demonstrate greater rate of cortical thinning. Although we were not powered to examine clusters of participants with varying levels of cortical thickness, leveraging a larger sample in future research may help clarify this finding. Nonetheless, our main finding that preserved baseline cognitive empathy predicted less thinning is in line with other studies in healthy individuals that demonstrate greater cortical integrity is related to empathy (26, 62, 63). Additionally, other studies have demonstrated that greater TPJ thinning in individuals with first-episode psychosis with persistent negative symptoms [often associated with reduced empathic abilities; (64, 65)] compared to controls (23). The lack of a relationship between right TPJ thinning and cognitive empathy performance in our healthy participants is not entirely unexpected given deriving relationships between brain structure and behavior in control groups requires larger samples to detect effects in variables with minimal variance (i.e., ceiling effects on cognitive empathy tasks and minor changes in cortical thickness in healthy individuals).

There are several limitations to this study that should be considered when interpreting these findings. First, cognitive empathy performance was measured at baseline, though not at the 24-month follow-up visit. Longitudinal assessment of cognitive empathy performance may be useful for determining if changes in TPJ thickness are related to changes in cognitive empathy, which would further establish the role of the TPJ in the cognitive empathy network and also raise implications for this region as an area of potential intervention (66). Second, changes in cortical thickness were measured over 2 years, which may not have been a sufficient enough time period to appreciate a greater degree of cortical thinning. Lastly, due to a small sample size and the requirement of imaging acquisition across 24 months, our findings may have limited generalizability. Beyond the identified limitations, our study had some notable strengths. First, to our knowledge, this is one of few studies that have measured brain structure longitudinally as it relates to cognitive empathy, in individuals with schizophrenia. Second, this study included a racially diverse sample in both the control and schizophrenia groups that may help enhance the generalizability of the findings. Lastly, the study used a well-validated measure of cognitive empathy (12, 33, 67).

Overall, our findings suggest that greater cognitive empathy may explain sustained integrity of the right TPJ in individuals with schizophrenia, suggesting a contributory substrate for the long-term maintenance of this process in psychosis. This study has implications for the progressive nature of brain structure changes in schizophrenia and how it may relate to behavior, namely cognitive empathy, and well as raises questions about a potential resilience mechanism. These findings also provide additional evidence for the unique role of the TPJ in cognitive empathy in schizophrenia.

## Data Availability Statement

The datasets presented in this study can be found in online repositories. The names of the repository/repositories and accession number(s) can be found here: http://schizconnect.org; NMorphCH.

## Ethics Statement

The studies involving human participants were reviewed and approved by Northwestern University Institutional Review Board. The patients/participants provided their written informed consent to participate in this study.

## Author Contributions

TK-R helped conceptualize the study, completed statistical analyses, and wrote the first draft. DC helped conceptualize the study, contributed to statistical analyses, and assisted with manuscript writing and editing. JP helped with study methods and contributed to manuscript editing. LW, VM, and JC contributed to the conceptualization of the study and assisted with manuscript editing. MS served as the principal investigator on this project, contributed to study conceptualization, oversaw all statistical analysis, and assisted with writing the first draft of the manuscript and subsequent manuscript editing. All authors approved the final manuscript and have made significant scientific contributions to this manuscript.

## Conflict of Interest

JC was compensated for service on a Data Safety Monitoring Board for Sunovion Pharmaceuticals. The remaining authors declare that the research was conducted in the absence of any commercial or financial relationships that could be construed as a potential conflict of interest.
